# From Remedy to Therapy: Confronting the Bioavailability Bottleneck in *Ganoderma lucidum* Translational Research

**DOI:** 10.3390/ph19081171

**Published:** 2026-07-27

**Authors:** Yujie Qu, Xinyu Zhao, Hongxin Liu, Rutong Ren, Wenran Qu, Shili Wang, Guohua Ma

**Affiliations:** 1 Medical School, Shandong Xiehe University, Jinan 250109, China; quyujie1@sdxiehe.edu.cn (Y.Q.); zhaoxinyu7@sdxiehe.edu.cn (X.Z.); liuhongxin@sdxiehe.edu.cn (H.L.); renrutong@sdxiehe.edu.cn (R.R.); quwenran@sdxiehe.edu.cn (W.Q.); wangshili@sdxiehe.edu.cn (S.W.); 2 Guangdong Provincial Key Laboratory of Applied Botany, South China Botanical Garden, The Chinese Academy of Sciences, Guangzhou 510650, China; 3 College of Life Science, University of the Chinese Academy of Sciences, Beijing 100049, China

**Keywords:** *Ganoderma lucidum*, bioavailability, triterpenoids, polysaccharides, nanotechnology, drug delivery, translational medicine

## Abstract

**Background/Objectives:** For over two millennia, *Ganoderma lucidum* has served as a traditional remedy, yet its translation into evidence-based therapy remains stymied by a persistent obstacle. Potent in vitro activities consistently fail to translate in vivo, a shortfall rooted in the poor systemic bioavailability of its signature triterpenoids and polysaccharides. We contend that the defining hurdle is no longer compound discovery but whether emerging formulation technologies genuinely overcome these barriers or merely sidestep them in ways that obscure the underlying physiology. **Methods:** We construct a structure–activity–formulation (SAF) prioritization framework. We critically survey a broad spectrum of delivery platforms—including lipid-based, polymeric, micellar, tellurium nanorod, and *G. lucidum*-derived vesicular systems—against their demonstrated capacity to improve oral exposure. Clinical assessments and regulatory considerations are integrated to anchor the analysis in translational reality. **Results:** Bioavailability has been systematically treated as a post hoc variable, even though the very structural features conferring bioactivity impose the steepest systemic barriers. To date, no *G. lucidum* nanoformulation has advanced to human trials, and the regulatory terrain for these complex natural-product nanomedicines remains uncharted. We distill three experimentally tractable propositions—testing lipid-mediated absorption, triple-helix conformational dependency, and co-delivery synergy—that offer discrete entry points for rigorous investigation. **Conclusions:** We argue that the field must pivot decisively from descriptive cataloging to hypothesis-driven, comparative investigation, anchored by reference-material standardization and proactive regulatory dialogue. Confronting the bioavailability bottleneck as a primary design parameter—rather than an ancillary nuisance—represents the most credible route from historical remedy to evidence-based therapeutic.

## 1. Introduction

For more than two millennia, *Ganoderma lucidum*—known as Lingzhi in China and Reishi in Japan—has occupied a revered place in East Asian pharmacopoeias. It has been prescribed to bolster vitality, calm the spirit, and prolong life [1]. Modern science has largely substantiated these ancient claims. Roughly 400 bioactive compounds have been identified, and an extraordinary spectrum of pharmacological activities documented [2]. Yet, this vast molecular arsenal has not translated into a single FDA-approved drug. The problem is not potency—it is delivery.

Despite the vast literature, *G. lucidum* remains a dietary supplement—not a drug—in the eyes of regulatory agencies and mainstream oncology. This failure is not for want of potent in vitro activity. The same ganoderic acids suppress cancer cell proliferation at micromolar concentrations, and the same β-glucans engage immune receptors with high affinity. Yet, both consistently fail to reproduce these effects in living organisms after oral administration—the route prescribed by traditional use. The obstacle is not insufficient intrinsic potency but a failure of systemic delivery [3,4].

This state of affairs reveals a central paradox. The lipophilic triterpenoids—lanostane skeletons with molecular weights exceeding 400 Da—exhibit aqueous solubility in the microgram-per-milliliter range. Their oral bioavailabilities generally fall below 20% in rodent models [3,4]. However, the absolute percentage is less important than the fact that peak plasma concentrations fall substantially short—often by an order of magnitude—of the micromolar levels required for direct in vitro cytotoxicity [5]. Ganoderic acid A (GAA), the most extensively studied triterpenoid, exemplifies this pattern. Its absolute oral bioavailability spans from 8.68% to 17.97% across rodent studies [6,7]. Its peak plasma concentrations (358.7–3010.4 ng/mL at 100–400 mg/kg [7]) hover roughly one order of magnitude below the micromolar concentrations required for direct cytotoxicity in vitro.

High-molecular-weight *G. lucidum* polysaccharides (GLPs) are water soluble, yet too large for efficient intestinal absorption. They act indirectly through gut-associated lymphoid tissue (GALT), eliciting local immunological effects without delivering the intact macromolecule to systemic targets [8]. The mechanistic underpinnings and translational implications of this GALT-centered pathway are examined in depth in Section 2.1. In vitro potency, then, does not predict in vivo efficacy at clinically achievable concentrations. Parenteral administration could bypass the intestinal epithelium, but for chronic adjuvant therapy and long-term immunomodulation, oral delivery remains the preferred route—for adherence and safety. For acute cancer treatment, rationally designed nanocarriers exploiting the enhanced permeability and retention (EPR) effect offer a complementary—albeit more invasive—approach. This review evaluates formulation strategies for both routes. We prioritize oral delivery for chronic applications, where patient adherence and safety almost entirely favor enteral regimens over repeated injections. This practical imperative renders the oral bioavailability problem a hard biological constraint, not a negotiable nuisance.

The field has largely circumvented this bottleneck rather than confronting it. For two decades, the field has operated under what we term a “phenomenological enumeration paradigm.” This is a relentless cataloging of new molecules and activities. Each report adds another entry to the already swollen list of what *G. lucidum* can do, yet rarely asks whether it can do any of these things well enough to help a single patient. Previous reviews, valuable as compendia, have compartmentalized chemistry, pharmacology, extraction, and formulation without interrogating their intersections [1,9,10]. Even the most recent systematic reviews provide comprehensive updates on the chemical composition and multifaceted health benefits of *G. lucidum*. Yet, they have continued to prioritize descriptive enumeration over the integrated structure–activity–formulation analysis that we argue is essential for translational success [11]. Few have asked whether extraction methods preserve or degrade structural features essential for bioactivity, or how GLP structure–activity relationships might guide the rational design of more potent derivatives. A 2025 systematic review acknowledged that variability in extraction methods and lack of standardized quality control remain fundamental obstacles to clinical translation [12]—but stopped short of asking why such variability persists.

The most consequential failure of *G. lucidum* translational research is not in oncology, where expectations are guarded, but in metabolic and inflammatory disease—the very indications for which the mushroom has been most aggressively promoted. Meta-analyses of randomized controlled trials have reached a pointed consensus: supplementation may shift selected biomarkers, but the evidence is too heterogeneous and underpowered to support any clinical guidance [13,14]. Yet, the same compounds, in animal models, deliver a strikingly different message. Systematic meta-analyses of in vivo studies confirm that ganoderic acids robustly suppress TNF-α, IL-1β, and IL-6 through coordinated inhibition of MAPK and TLR4/NF-κB signaling [15]. That this mechanistic rigor evaporates in human trials is not a failure of pharmacology—it is a failure of pharmacokinetics. At the doses tested, unformulated oral extracts cannot sustain the systemic concentrations required to engage these targets. The null results, therefore, do not condemn the molecule. They condemn the mode of administration. In doing so, they precisely define the bioavailability bottleneck that our Structure–Activity–Formulation (SAF) framework is designed to overcome.

This paradigm endures not from scientific necessity but from disciplinary fragmentation and publication incentives that reward novel compound discovery over rigorous pharmacokinetic characterization. Natural product chemistry and pharmaceutical sciences operate in parallel silos: the former cataloging what the mushroom contains, the latter occasionally attempting to formulate it. This separation has allowed the bioavailability question to be perpetually deferred.

We propose an alternative. The path from remedy to therapy requires confronting the bioavailability bottleneck directly—not as an afterthought or a “formulation challenge” to be outsourced to the final stage. Instead, it must be the central problem organizing the entire translational enterprise. This demands reassessing which compounds merit development based on pharmacokinetic and pharmacodynamic criteria. It also requires evaluating whether advanced extraction technologies genuinely improve bioavailability or merely increase yield. We need systematic comparisons of nanocarrier platforms against translational performance metrics rather than their own internal benchmarks. Finally, we must appraise the regulatory and clinical barriers that have kept every *G. lucidum* nanoformulation out of patients [2].

The most pressing challenge in *G. lucidum* research is not discovering new compounds or enumerating additional activities, but resolving a fundamental delivery problem. This review advances three interconnected arguments. First, the structural features that confer pharmacological activity impose severe pharmacokinetic constraints—an SAF framework the field has not systematically addressed. Second, enthusiasm for advanced extraction and nanocarrier platforms has outpaced critical evaluation of whether these approaches genuinely solve the bioavailability problem or merely add technical sophistication without proportional therapeutic gain. Third, the clinical evidence base—anchored by the Cochrane review—reveals that no *G. lucidum* nanoformulation has reached human trials. Moreover, the regulatory pathway for complex natural product nanomedicines remains undefined [2,16].

By focusing on these intersections rather than exhaustive enumeration, we provide a roadmap for moving beyond description toward hypothesis-driven investigation. We propose three testable hypotheses. First, lipid nanoparticle encapsulation can increase GAA oral bioavailability by at least five-fold. Second, extraction conditions preserving polysaccharide triple-helix conformation will yield superior immunomodulatory activity even at lower yields. Third, co-delivery of triterpenoids and GLPs within a single nanocarrier can produce synergistic effects exceeding those of separate formulations. The mechanistic rationale and evidence for each hypothesis are elaborated below. Only by confronting the bioavailability bottleneck directly—with rigor, hypothesis-driven experimentation, and a willingness to abandon the comfortable paradigm of enumeration—can *G. lucidum* evolve from a revered remedy into a credible therapeutic.

## 2. The Chemistry of Promise: Bioactive Constituents and Their Structural Determinants

### 2.1. GLPs: Structure, Recognition, and the Immunological Bridge

GLPs are the most abundant bioactive macromolecules in *G. lucidum*. They constitute 10–50% of fruiting-body dry matter [17]. Their immunomodulatory properties are well established, yet the relationship between structural features and biological activity remains incompletely understood. This limitation has direct implications for formulation design.

Structurally, the dominant motif among bioactive GLPs is a β-(1→3)-D-glucan backbone with β-(1→6)-linked side chains [18,19]. This configuration is critical for recognition by Dectin-1, the primary β-glucan receptor on macrophages and dendritic cells. GLP preparations are notably heterogeneous. Molecular weights span roughly 3 to >700 kDa. Monosaccharide composition varies, with glucose, galactose, mannose, and arabinose present in differing ratios. Branching patterns diverge substantially [8,20]. A purified β-D-glucan from fruiting bodies (GLP20) has a β-(1→3)-linked backbone with (1→6)-β-D-glucopyranosyl side branches on every third residue. Spore-derived GLPs share comparable backbones but exhibit more complex branching [21].

A novel galactose-rich polysaccharide (GLP-1b, 16.79 kDa) from *G. lucidum* features an α-1,6-linked galactan backbone. It carries C-2/C-3 acetylation and complex branching with diverse terminal residues [22]. This polysaccharide enhances macrophage function through Toll-like receptor 4 (TLR4)-dependent activation of nuclear factor kappa-B (NF-κB) and mitogen-activated protein kinase (MAPK) signaling. It increases nitric oxide (NO) production and pro-inflammatory cytokine secretion [22]. As discussed later in Section 4.5, however, this signaling outcome is not invariant. It depends critically on the physical form of delivery.

Functionally, the relationship between molecular weight and bioactivity is non-monotonic and context dependent. High-molecular-weight GLPs (>300 kDa) generally exhibit more potent immunomodulatory effects. They crosslink multiple Dectin-1 receptors more effectively, forming signaling-competent clusters [23,24]. Lower-molecular-weight fractions (8–50 kDa) show superior antioxidant activity, presumably owing to more efficient cellular penetration and free-radical scavenging [25]. A 44.4 kDa polysaccharide enhanced antitumor activity in Lewis lung carcinoma-bearing mice. It increased CD4^+^ and CD8^+^ T cell proportions in splenic tissue [24]. By contrast, an 8.9 kDa fraction more strongly regulated oxidative stress markers. It decreased reactive oxygen species (ROS) and malondialdehyde while increasing antioxidant enzyme activities [25]. A polysaccharide purified from Taishan *G. lucidum* (GLP-2, 8495 Da) is composed of galactose, fucose, mannose, and glucose. Its main chain has →6) α-D-Galp-(1→ and →4) α-D-Galp-(1→ sequences. This GLP-2 preparation alleviated atopic dermatitis in mice by increasing the relative abundance of *Prevotella* and *Ruminococcus*. It elevated short-chain fatty acid levels and inhibited ROS through heme oxygenase-1 (HO-1) and nuclear factor erythroid 2-related factor 2 (NRF2) pathways [26]. This inverse relationship—between immunomodulatory and antioxidant activities—presents a formulation dilemma the field has not systematically addressed. A single GLP preparation cannot optimize both activities, yet most studies treat GLPs as uniform entities.

Monosaccharide composition further modulates activity. GLPs with higher mannose and glucose content are associated with enhanced antioxidant and anti-inflammatory effects [25,27]. A polysaccharide-peptide complex (GLPP) containing higher molar ratios of mannose and glucose alleviated collagen-induced arthritis symptoms more effectively than preparations with different compositions [27]. Mannose-rich domains may facilitate recognition by the mannose receptor (CD206) in addition to Dectin-1. This would broaden the immunological response profile. This hypothesis, however, requires direct experimental validation.

Beyond primary sequence, the triple-helix conformation—a feature of many bioactive β-glucans—appears to be another determinant of activity. This higher-order structure is stabilized by interchain hydrogen bonds. It may facilitate multivalent receptor interactions and enhance signaling potency [28]. Yet, the triple helix is susceptible to denaturation under extraction conditions involving elevated temperatures or prolonged sonication. This raises the possibility that conventional processing methods inadvertently reduce bioactivity while increasing yield [29].

This structural heterogeneity carries a strategic implication that the field’s fixation on systemic bioavailability has largely obscured. For high-molecular-weight GLPs (>300 kDa), biophysical exclusion from transepithelial transport is not a barrier to be surmounted—it is a biological datum to be exploited. The therapeutic bridge for these macromolecules lies not in plasma concentration but in the gut microbiota–GALT axis. They act as prebiotic modulators, enriching short-chain fatty acid (SCFA)-producing genera such as *Prevotella* and *Ruminococcus* [27,30]. They also engage pathogen-associated molecular pattern (PAMP) receptors on intestinal immune cells without ever crossing the epithelial barrier. Controlled in vitro digestion and fermentation studies have shown that *G. lucidum* increases propionate, butyrate, and valerate production during colonic fermentation [31]. Broader reviews of edible fungal bioactives—β-glucans and ganoderic acids included—have converged on a parallel mechanistic logic: improving intestinal barrier function and immune homeostasis through microbiota modulation, SCFA enhancement, and pathogen colonization inhibition [32].

The caveat is that nearly all evidence for this framework rests on preclinical models. The translational gap between murine microbiome shifts and human therapeutic outcomes has not been systematically closed. The spatiotemporal dynamics of fungal polysaccharide fermentation in the human colon—let alone their downstream effects on systemic immunity—remain poorly resolved [32]. This uncertainty does not invalidate the GALT-centered strategy. It simply requires that we calibrate expectations realistically. Not every GLP constituent requires nanocarrier formulation. The appropriate delivery strategy must be dictated by molecular weight, site of action, and therapeutic endpoint. The SAF framework rests on precisely this premise: formulation decisions should flow from structural–pharmacological logic, not from the reflexive application of nanotechnology to every molecule with laboratory activity.

GLP bioactivity is not a monolithic property. It is an emergent consequence of molecular weight distribution, monosaccharide composition, glycosidic linkage pattern, and conformational state. This structural complexity has profound implications for translational development. Standardization of GLP preparations requires control over all these parameters. Yet, most studies use crude extracts with poorly characterized polysaccharide profiles. Without such standardization, comparisons across studies—and, more critically, across clinical preparations—remain inherently unreliable.

### 2.2. Triterpenoids: Potency at the Price of Poor Solubility

If GLPs represent the immunological arm of *G. lucidum*’s pharmacology, triterpenoids constitute its direct cytotoxic and anti-inflammatory component. Over 380 triterpenoids have emerged from phytochemical profiling. They are predominantly lanostane-type tetracyclic structures with varying oxidation states and functional group modifications [30,33]. The ganoderic acids—featuring hydroxyl, carboxyl, or keto substitutions on the tetracyclic scaffold—constitute the most extensively studied subclass. They have molecular weights of 400–600 Da and pronounced lipophilicity [3,10].

GAA exemplifies this challenge. GAA exerts anti-inflammatory, antioxidant, and antitumor effects through NF-κB, Janus kinase/signal transducer and activator of transcription (JAK/STAT), TLR4, and MAPK pathways [34]. Its antitumor activity—inhibiting proliferation, inducing apoptosis, and suppressing angiogenesis—is among the best-characterized and most translationally relevant of the ganoderic acids [35,36]. Yet, its pharmacokinetic profile exposes the fundamental obstacle. Rapid absorption followed by rapid elimination yields low overall bioavailability [34]. Reported oral bioavailability values range from 8.68% [6] to 10.38–17.97% [7]. This spread is likely attributable to differences in dosing vehicles, analytical protocols, or animal physiological states.

Critically, whether one adopts the lower or higher estimate, the maximum plasma concentration (Cmax: 358.7–3010.4 ng/mL at 100–400 mg/kg [7]) falls approximately one order of magnitude below the micromolar concentrations required for direct cytotoxicity in vitro. This exposure–efficacy gap, rather than the precise bioavailability percentage, defines the true translational bottleneck. GAA also penetrates the blood–brain barrier, with brain-to-plasma ratios of 0.05–0.18. This indicates limited but measurable central nervous system (CNS) exposure [6].

The compound’s lipophilicity, while facilitating membrane permeation, also promotes hepatic extraction and metabolic clearance. This is a classic first-pass liability. In healthy volunteers, the pharmacokinetic profiles of GAA and ganoderic acid F (GAF) under fasting conditions are characterized by rapid absorption (Tmax ≈ 30 min) and a short elimination half-life (<40 min). Food significantly decreased Cmax and delayed Tmax of GAF but did not affect the extent of GAA absorption. By contrast, concomitant food intake markedly impeded both the rate and extent of GAF absorption [37].

The bioavailability challenge extends across the triterpenoid class. Pharmacokinetic studies of triterpenoid-enriched fractions have documented maximum plasma concentrations in the nanogram-per-milliliter range following oral administration at gram-equivalent doses [4]. A sensitive liquid chromatography-tandem mass spectrometry (LC-MS/MS) method was developed for the simultaneous determination of ganoderic acid D (GAD) and its main metabolite (ganoderic acid B) in rat plasma. This method demonstrated that the absolute bioavailability of GAD increased from 22% to 70% after formulation as GAD-loaded solid lipid nanoparticles (SLN). Cmax increased from 107.2 to 1555.6 ng/mL, and Tmax shortened from 2.0 h to 0.3 h [38]. The first high-performance liquid chromatography (HPLC) method for the quantitative determination of four major triterpenoids—ganoderic acids GAC_2_, GAB, GAK (ganoderic acid K), and GAH (ganoderic acid H)—in rat plasma after oral administration of *G. lucidum* extract achieved extraction recoveries of 67–95%. Intra- and inter-day precision deviations fell below 10%. This represented the first report on systemic exposure of multiple ganoderic acids [39]. An ultra-performance liquid chromatography quadrupole time-of-flight mass spectrometry (UPLC-Q-TOF/MS)-based study of *G. lucidum* ethanol extract in tumor-bearing nude mice examined the distribution of triterpenoids across eight organs, plasma, and tumors. It identified 42 prototype compounds and 24 metabolites after six weeks of administration. GAA, GAB, GAC_1_, GAF, and ganoderic acid H (GAH) were the most widely distributed [40]. *G. lucidum* triterpenes—including GAA, GAC_2_, GAD, GAF, ganoderic acid G (GAG), and GAH—exhibit rapid absorption and sustained tissue retention. They ameliorate age-related cognitive impairment via regulating *Eubacterium lentum*-mediated serine metabolism [41]. These findings suggest some degree of systemic exposure. Yet, the concentration gap between systemic exposure and in vitro efficacy underscores the magnitude of the delivery problem.

The structural features that confer pharmacological activity are precisely those that impose pharmacokinetic constraints. The tetracyclic lanostane skeleton and multiple oxygenated substituents create a molecule too lipophilic for aqueous formulation yet too polar for optimal passive membrane permeation. Structurally similar lanostane-type triterpenoids exhibit poor intestinal permeability in Caco-2 cell models [42]. This “biphasic” character places triterpenoids in the Biopharmaceutics Classification System (BCS) Class II or IV, indicating low solubility with either high or low permeability. For such compounds, formulation strategies that enhance dissolution or protect against metabolic degradation are not optional enhancements. They are essential prerequisites for therapeutic activity. Encapsulation of ganoderic acids into zein-chitosan nanoparticles was necessary to overcome their low oral bioaccessibility. This achieved superior hepatoprotective effects compared to non-encapsulated forms [43].

**Figure 1:**
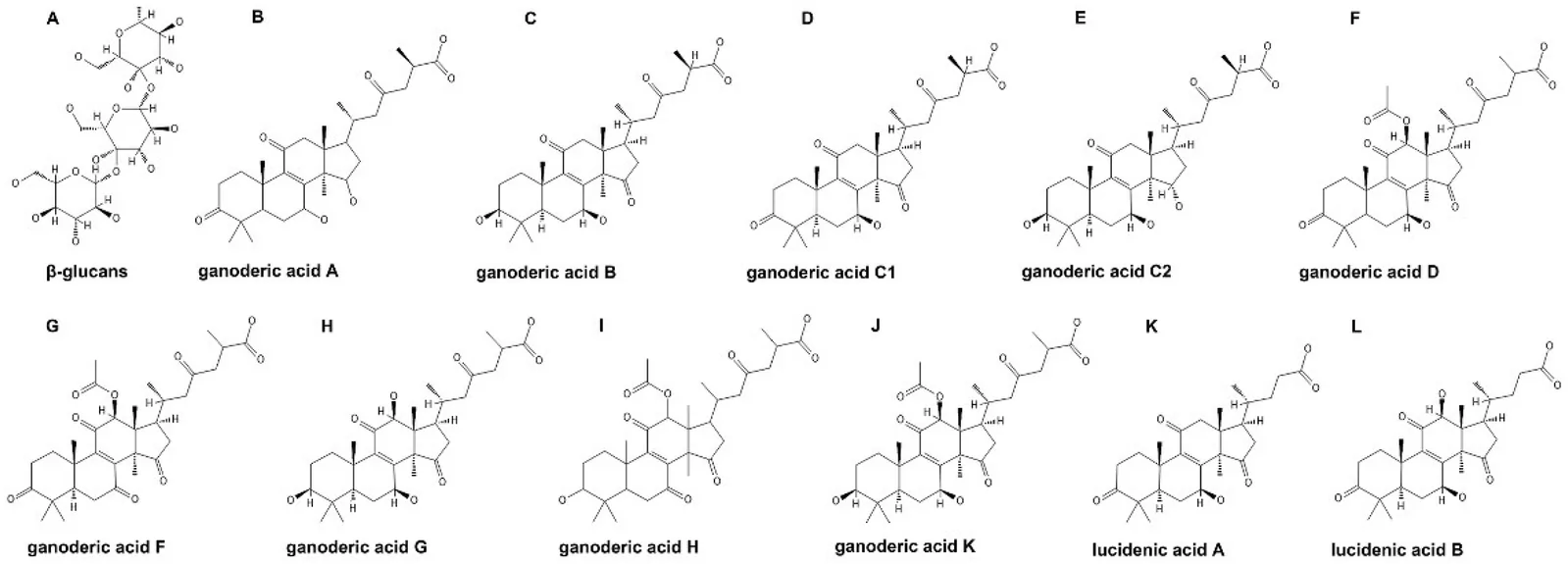
Chemical structures of major *G. lucidum* bioactives. (**A**) β-(1→3)-D-glucan backbone with β-(1→6)-branches, the core motif of immunomodulatory GLPs (triple-helix conformation stabilized by hydrogen bonds). (**B**–**L**) Lanostane-type triterpenoids (ganoderic acids (**B**–**J**) and lucidenic acids (**K**,**L**)). The number/position of –OH, –COOH, and C=O substituents dictate LogP, aqueous solubility, and metabolic stability—directly accounting for the low oral bioavailability (e.g., GAA: 8.68–17.97%) and subtherapeutic systemic exposure that define the translational bottleneck.

The diversity of the triterpenoid pool complicates standardization. Different ganoderic acids exhibit distinct solubility profiles, metabolic fates, and bioactivities [33,44]. A crude triterpenoid extract containing dozens of compounds will have pharmacokinetic properties that reflect the average of its constituents, not the optimal properties of any single component. This compositional heterogeneity makes it difficult to establish meaningful quality control standards. It also obscures attribution of therapeutic effects to specific compounds. Until the field moves toward purified, well-characterized triterpenoid preparations—or at minimum, extracts with defined compositional profiles—the translational gap will persist. Figure 1 presents the chemical structures of representative *G. lucidum* bioactives—the β-glucan backbone of GLPs and major lanostane-type triterpenoids—and illustrates how their distinct structural features dictate the physicochemical properties that constrain oral bioavailability. The available pharmacokinetic data for individual triterpenoids, compiled in Table 1, reveal a consistent pattern. Oral exposure falls orders of magnitude below the concentrations required for direct in vitro activity—a quantitative reality the field has systematically neglected.

## 3. Extraction Technologies: Optimizing Yield or Preserving Activity?

The choice of extraction method is the first critical decision in the translational pipeline. It determines not only the quantity of recovered compounds but also their structural integrity and biological activity. Advanced technologies—ultrasonic-assisted, microwave-assisted, supercritical fluid, and enzyme-assisted methods—have proliferated. Each promises superior yields and reduced processing times [45]. Yet, enthusiasm for these technologies has often outpaced critical evaluation of whether increased yield translates to improved bioactivity or bioavailability.

### 3.1. Conventional Methods: Established but Inefficient

Hot water extraction (HWE) and ethanol maceration remain the standard methods for polysaccharide and triterpenoid recovery, respectively. These reflect traditional preparation practices. Typical HWE conditions (95 °C, 2 h) yield approximately 1.5% GLPs from fruiting bodies. Ethanol maceration (30 °C, 6 h) yields approximately 0.6% triterpenoids [46]. These yields are low, but the methods are simple, inexpensive, and scalable. These attributes have sustained their use despite more efficient alternatives.

Prolonged heating, however, can degrade thermolabile compounds and modify polysaccharide conformation [28]. The high temperatures required for HWE may denature the β-glucan triple-helix structure. This potentially reduces immunomodulatory potency while increasing polysaccharide yield [29]. Alkaline extraction achieves higher β-glucan yields (up to 9.3% dry weight). Yet, it may hydrolyze acid-sensitive glycosidic bonds and alter the fine structure of the polysaccharide backbone [47]. Conventional methods, adequate for traditional use, may therefore be suboptimal for producing extracts suitable for modern pharmaceutical development.

### 3.2. Advanced Technologies: Higher Yield, Unresolved Questions

Ultrasonic-assisted extraction (UAE) employs mechanical effects and cavitation to disrupt cell walls and enhance mass transfer. Optimized UAE conditions—40 min, 100 W ultrasonic power, and 89.5% ethanol—recovered 4.9% ± 0.6% of extractable material. Triterpenoid and total phenolic contents reached 435.6 ± 21.1 mg/g and 106.6 ± 16.2 mg/g of extract, respectively [48]. Prolonged ultrasonic treatment, however, can degrade GLPs through chain scission. This reduces molecular weight and potentially alters bioactivity [20]. No study has systematically characterized this yield-structure trade-off for most GLP preparations.

One study applied response surface methodology coupled with artificial neural network-genetic algorithm modeling to optimize UAE parameters. The optimal conditions—320 W, 74% ethanol, 61 mL/g liquid-solid ratio, 69 min—produced 4.61 ± 0.08 mg triterpenoids and 4.53 ± 0.09 mg phenolics per gram of *G. lucidum* [49]. UPLC-Q-TOF MS/MS analysis further resolved 20 triterpenoids (9 newly reported) and 8 phenols (7 newly reported) in the optimized extracts [49]. Whether this yield improvement translates to enhanced bioactivity remains untested.

For speed, microwave-assisted extraction (MAE) offers processing times as short as 1.5 min at 800 W with 65% ethanol. It achieves polysaccharide and triterpenoid yields of 13.08 mg glucose/g and 9.15 mg ursolic acid/g, respectively [50]. The rapid heating and pressure buildup can fracture cell walls more efficiently than conventional methods. Yet, the high energy input raises concerns about thermal degradation of sensitive compounds. Ultrasonic-microwave assistance (UMAE) increases polysaccharide yield by 115.6% over HWE and 27.7% over UAE alone [51]. Impressive as these yield improvements are, they do not address the more fundamental question. Does the extracted polysaccharide retain the structural features—molecular weight distribution, branching pattern, conformational state—that determine biological activity?

Supercritical CO_2_ extraction (SCE) operates at high pressure (43 MPa) and moderate temperature (54.8 °C). It offers solvent-free processing and has been applied primarily to triterpenoid recovery [52]. The absence of organic solvents and the low operating temperature preserve labile compounds and prevent oxidation of unsaturated fatty acids. High capital cost and technical complexity, however, have limited its adoption. Yields (1.56 mg triterpenoids/100 g) remain modest.

### 3.3. A Critical Gap: Bioactivity as the Missing Outcome Variable

Systematic comparisons that include bioactivity as an outcome are conspicuously absent from the extraction literature. Most studies report yield as the primary endpoint, with occasional measurements of antioxidant capacity or total phenolic content. Few ask whether extracts prepared by different methods differ in immunomodulatory, antitumor, or anti-inflammatory potency in relevant biological systems. Even fewer ask whether such differences correlate with structural features of the extracted compounds. The field has largely outsourced the definition of “quality” to industrial economics rather than pharmacological criteria.

This gap carries practical consequences, not just academic ones. Extraction methods optimized for maximum yield may degrade the structural features essential for bioactivity. The net therapeutic effect could remain unchanged or even diminish. The literature is replete with “optimized extraction” studies that serve industrial cost-reduction goals. They contribute little to mechanistic understanding of what makes *G. lucidum* extracts therapeutically effective. Conversely, if conventional methods preserve bioactivity at lower yields, the appropriate strategy might be process refinement rather than technology replacement. The field’s current fixation on yield—driven by commercial imperatives and publication metrics—obscures more fundamental questions about the relationship between extraction conditions and therapeutic potential.

We propose a “bioactivity-guided extraction” framework: optimize conditions for maximum biological activity in relevant assays, not for maximum yield. This would require integrating bioassay data into the optimization process from the outset. It cannot be treated as a secondary characterization step. It would also necessitate standardized bioassays that reliably proxy for in vivo activity. This is a formidable challenge given the complexity of the biological responses that *G. lucidum* extracts elicit. As summarized in Table 2, the extraction literature offers abundant yield data. Yet, it provides remarkably little evidence on whether the resulting extracts retain the structural features required for biological activity. This discrepancy underscores the need for bioactivity-guided process development.

## 4. Nanotechnology: Promise, Progress, and Unresolved Questions

The bioavailability bottleneck stands as the central obstacle to *G. lucidum* translational development. Nanotechnology offers the most plausible strategy for overcoming it. Nanocarriers can protect labile compounds from degradation, enhance aqueous solubility, and prolong circulation time. They can also exploit the enhanced permeability and retention (EPR) effect for passive tumor targeting [53]. Several nanocarrier platforms—lipid nanoparticles, polymeric nanoparticles, polymeric micelles, and metallic nanoparticles—have been explored for *G. lucidum* delivery. Preclinical results are promising [54,55].

Yet, the gap between preclinical promise and clinical reality remains vast. No *G. lucidum* nanoformulation has entered human trials. The regulatory path remains undefined [2]. This section evaluates the evidence for different nanocarrier platforms and identifies the translational barriers that have prevented clinical progress. We also examine emerging strategies—including GLP-based tellurium nanorods and *G. lucidum*-derived nanovesicles—that represent new directions in nanotherapeutic development.

### 4.1. Lipid Nanoparticles: Clinically Advanced but Under-Explored for G. lucidum

Lipid nanoparticles (LNPs)—including liposomes, SLNs, and nanostructured lipid carriers (NLCs)—are the most clinically advanced nanocarrier platform. Multiple FDA-approved products, including Doxil/Caelyx and Vyxeos, use this platform [56,57]. LNPs offer biocompatibility, biodegradability, and high encapsulation efficiency for lipophilic compounds. These properties make them natural candidates for triterpenoid delivery. For translational readiness, LNPs are the platform of choice for ganoderic acid delivery, given their established regulatory pathways and clinical track record.

For *G. lucidum* applications, the evidence base for LNPs remains limited but suggestive. A zein-chitosan nanoparticle formulation loaded with ganoderic acids achieved high encapsulation efficiency (92.68%), small particle size (177.20 nm), and favorable surface charge (+29.53 mV) [43]. In an alcohol-induced liver injury mouse model, ganoderic acid-loaded nanoparticles (GA-NPs) significantly improved liver metabolic function and reduced oxidative stress. They also ameliorated intestinal microbiota dysbiosis. These effects were superior to non-encapsulated ganoderic acids [43]. Ganoderic acid-loaded solid lipid nanoparticles (GA-SLNs) ameliorated D-galactosamine-induced hepatotoxicity in Wistar rats. Treated groups showed restored serum hepatic markers and antioxidant enzyme levels compared to free ganoderic acid [58]. GAA suppresses autophagy by regulating the circFLNA/miR-486-3p/CYP1A1/XRCC1 axis. This strengthens the sensitivity of lung cancer cells to cisplatin [59]. Liposomal formulations of GLPs have also shown promise. GLP liposomes enhanced immunomodulatory activity in a porcine circovirus model, increasing antibody titers and cytokine production [60]. GLPs were also shown to potentiate mRNA-LNP efficacy by synergizing oxidative stress mitigation with innate immune modulation. GLP-LNP formulations alleviated intracellular oxidative stress by elevating glutathione and superoxide dismutase while reducing malondialdehyde. This indicates restored redox homeostasis [61].

The primary limitation of the LNP literature for *G. lucidum* is the narrow range of formulations and indications explored. Most studies use single formulations in single disease models. They lack systematic optimization of lipid composition, surface modification, or dosing regimen. Head-to-head comparisons with other nanocarrier platforms are virtually absent. This prevents evidence-based platform selection. The translational trajectory of LNPs for *G. lucidum* thus remains at an early stage.

### 4.2. Polymeric Nanoparticles: Controlled Release with Manufacturing Challenges

Polymeric nanoparticles (PNPs)—particularly those formulated with biodegradable polyesters such as poly(lactic-co-glycolic acid) (PLGA) or natural polymers such as chitosan—offer complementary advantages. They provide controlled release kinetics, high physical stability, and versatile surface functionalization for active targeting [62,63]. In the context of *G. lucidum*, PNPs have been explored primarily for polysaccharide delivery. From a translational readiness perspective, PNPs are promising but manufacturing-limited. Their clinical translation has lagged behind LNPs because of scalability challenges [64].

GLP-based nanoparticles have demonstrated antitumor efficacy in preclinical models. GLP nanoparticles prepared by self-assembly showed cytotoxic effects on tumor cells and growth-promoting effects on spleen cells [65]. pH-sensitive nanoparticles based on GLP-methotrexate conjugates achieved programmable release of multiple drugs. They showed enhanced antitumor activity in xenograft models [66,67]. GLP-stabilized selenium nanoparticles (GLP-SeNPs), synthesized using GLPs (Mw = 983.96 kDa) as a stabilizer, prevented metabolic-associated fatty liver disease (MAFLD). They targeted ferroptosis through activation of the NRF2-mediated glutathione-glutathione peroxidase 4 (GSH-GPX4) pathway, acyl-CoA synthetase long-chain family member 4 (ACSL4)-mediated iron pathway, and ACSL4-mediated lipid metabolism [68]. Chitosan-polysaccharide nanoparticles containing GLPs induced apoptosis in PC3 cells by upregulating the pro-apoptotic *BAX* gene 4.6-fold and downregulating the anti-apoptotic *BCL2* gene to 0.64-fold. This highlights their potential for prostate cancer therapy [69].

The translational challenge for PNPs lies in manufacturing scalability and reproducibility. Achieving consistent, homogeneous large-scale batches remains a key limitation [64]. The batch-to-batch variability inherent in natural polymer extraction compounds this challenge. GLP preparations vary in molecular weight distribution and composition, which affects nanoparticle formation, drug loading, and release kinetics. Without standardized GLP starting materials, reproducible PNP manufacturing is difficult to achieve.

### 4.3. Polymeric Micelles: Solubilization with Instability Concerns

Polymeric micelles, formed by self-assembly of amphiphilic block copolymers, offer excellent solubilization of poorly soluble drugs. They have been explored for triterpenoid delivery [70,71]. Their small size (10–100 nm) and core–shell structure make them attractive for oral and intravenous delivery. Micelles are conceptually attractive but instability-prone. Dilution-dependent disassembly upon administration raises concerns about premature drug release [70].

The literature on polymeric micelles for *G. lucidum* delivery is sparse but conceptually promising. Micellar formulations enhance the solubility and bioavailability of ganoderic acids [38]. For oral delivery, however, susceptibility to gastrointestinal degradation and challenges in achieving sufficient intestinal residence time represent additional barriers.

### 4.4. Metallic Nanoparticles: Intrinsic Activity with Safety Questions

Silver nanoparticles synthesized using *G. lucidum* extracts (GL-AgNPs) represent a distinct category. The mushroom extract serves as both reducing and stabilizing agent for the metallic core [72,73]. GL-AgNPs have demonstrated antimicrobial activity against drug-resistant bacteria [74]. Metallic nanoparticles are intriguing but raise safety concerns. Long-term metal accumulation and off-target toxicity remain significant barriers [75]. Their limited biodegradability further complicates development. For most therapeutic applications, the risk-benefit profile is unlikely to favor metallic nanoparticles over biodegradable alternatives.

### 4.5. Emerging Nanotherapeutic Strategies

GLP-based multifunctional tellurium nanorods (GLP-LU-TeNRs)—where ‘GLP’ denotes the crude polysaccharide fraction rather than a specific purified isolate—were developed using GLPs and luteolin to regulate one-dimensional tellurium growth. These nanorods have an aspect ratio of 5.8 and a photothermal conversion efficiency of 30.9%. Upon 808 nm near-infrared irradiation, GLP-LU-TeNRs inhibited cancer cell proliferation by up to 22.8% in vitro (150 μg/mL). In vivo, they suppressed tumor growth and metastasis by 36.1% and 66.7%, respectively, compared to non-laser controls. The GLP component upregulated NO production and promoted dendritic cell maturation, initiating antitumor immune responses. This platform integrates immunotherapy, chemotherapy, and photothermal therapy within a single nanocarrier [76].

*G. lucidum*-derived nanovesicles (GLNs)—a novel class of fungus-origin nanotherapeutics distinct from the soluble GLPs discussed above—when orally administered in a doxorubicin-induced acute aging mouse model, mitigated multiorgan injury. This was evidenced by reduced serum ALT and AST levels [77]. GLNs attenuated oxidative stress by decreasing malondialdehyde through activation of the Nrf2/HO-1 antioxidant pathway. They suppressed senescence-associated β-galactosidase activity and downregulated p21 and p16. Mechanistically, GLNs inhibited NF-κB activation, reduced pro-inflammatory cytokines (IL-6, TNF-α), and upregulated IL-10. These findings position GLNs as a safe, orally administrable nanoplatform with antioxidant, antisenescent, and anti-inflammatory activities [77].

A water-soluble glucan (WSG) derived from *G. lucidum* was formulated into a dissolvable microneedle patch (MN-WSG). This patch suppressed melanoma cells by targeting TGFβ receptor-mediated Snail and Twist pathways. In vivo studies showed that WSG suppressed melanoma growth in B16F10-bearing mice [78]. This represents a novel transdermal delivery approach for GLP-based therapeutics. A polysaccharide designated EPGLa (*G. lucidum* polysaccharide a) (derived from *G. lucidum*) alleviated non-alcoholic fatty liver disease by promoting the growth of beneficial gut bacteria to repair the intestinal barrier against lipopolysaccharides. It also enhanced short-chain fatty acid production to activate the AMPK pathway, while simultaneously suppressing the TLR4/NF-κB/MAPK inflammatory cascade [79].

The mechanistic contrast between soluble GLPs and GLN vesicles is illuminating. Soluble GLPs activate NF-κB, driving pro-inflammatory, tumoricidal responses [23]. GLN vesicles do the opposite—they suppress NF-κB to exert anti-senescent, anti-inflammatory effects [77]. This is not a contradiction; it is a validation of the SAF framework’s core premise. The physical form and supramolecular assembly of a delivery system fundamentally alter receptor engagement kinetics and subcellular trafficking, re-routing the functional output of the same NF-κB axis. Soluble β-glucan crosslinks Dectin-1 into signaling-competent clusters; vesicular encapsulation delivers a supramolecular cargo that favors redox modulation over inflammatory transcription. This context-dependent bifunctionality is not a liability but a therapeutic opportunity—contingent on aligning formulation design with the intended functional direction.

While these results are encouraging, several caveats warrant attention. First, the photothermal therapy data for GLP-LU-TeNRs were obtained under optimized laser conditions that may not be clinically feasible. Second, the long-term safety of tellurium accumulation has not been assessed. Third, the oral bioavailability of GLNs in humans remains speculative. The field should treat these emerging platforms as proof-of-concept rather than ready-for-clinical-translation solutions. These platforms force a fundamental question: what is the optimal strategy for *G. lucidum* nanotherapeutic development? GLP-LU-TeNRs and GLNs differ fundamentally from conventional nanocarrier formulations. They are not simply delivery vehicles for purified compounds but complex assemblies in which the mushroom-derived material serves both therapeutic and structural functions. This blurring of the boundary between “active ingredient” and “excipient” creates new regulatory and manufacturing challenges while potentially offering synergistic benefits.

### 4.6. Comparative Analysis: What the Data Do and Do Not Show

A question the field has largely avoided is whether every bioactive compound from *G. lucidum* requires nanocarrier formulation. Water-soluble GLPs exert immunomodulatory effects through gut-associated lymphoid tissue—a route that does not necessarily require systemic bioavailability. The uncritical application of nanotechnology to all compound classes risks adding complexity and cost without proportional therapeutic gain. Future studies should establish clear criteria for when nanotechnology is genuinely required versus when it merely adds technical sophistication without clinical benefit.

The most striking feature of the nanocarrier literature for *G. lucidum* is the absence of systematic comparative studies. No study has directly compared different nanocarrier platforms loaded with the same well-characterized *G. lucidum* extract or purified compound, using identical endpoints and animal models. This absence prevents evidence-based platform selection and obscures the relative advantages of each approach. This lacuna reflects a deeper problem: the field’s fragmentation into platform-specific silos, with researchers committed to particular technologies rather than to identifying the optimal solution for a given therapeutic problem.

What limited comparative data exist come from studies using other drugs. A head-to-head comparison of PLGA nanoparticles and SLNloaded with the same poorly soluble drug revealed a trade-off. PNPs showed higher physical stability but lower encapsulation efficiency (54.3% versus 100%). They achieved higher oral bioavailability (12.7% versus 4.4%) with a shorter time to peak concentration [80]. No single platform universally outperforms all others. The optimal choice depends on the specific drug, administration route, and therapeutic indication.

**Figure 2:**
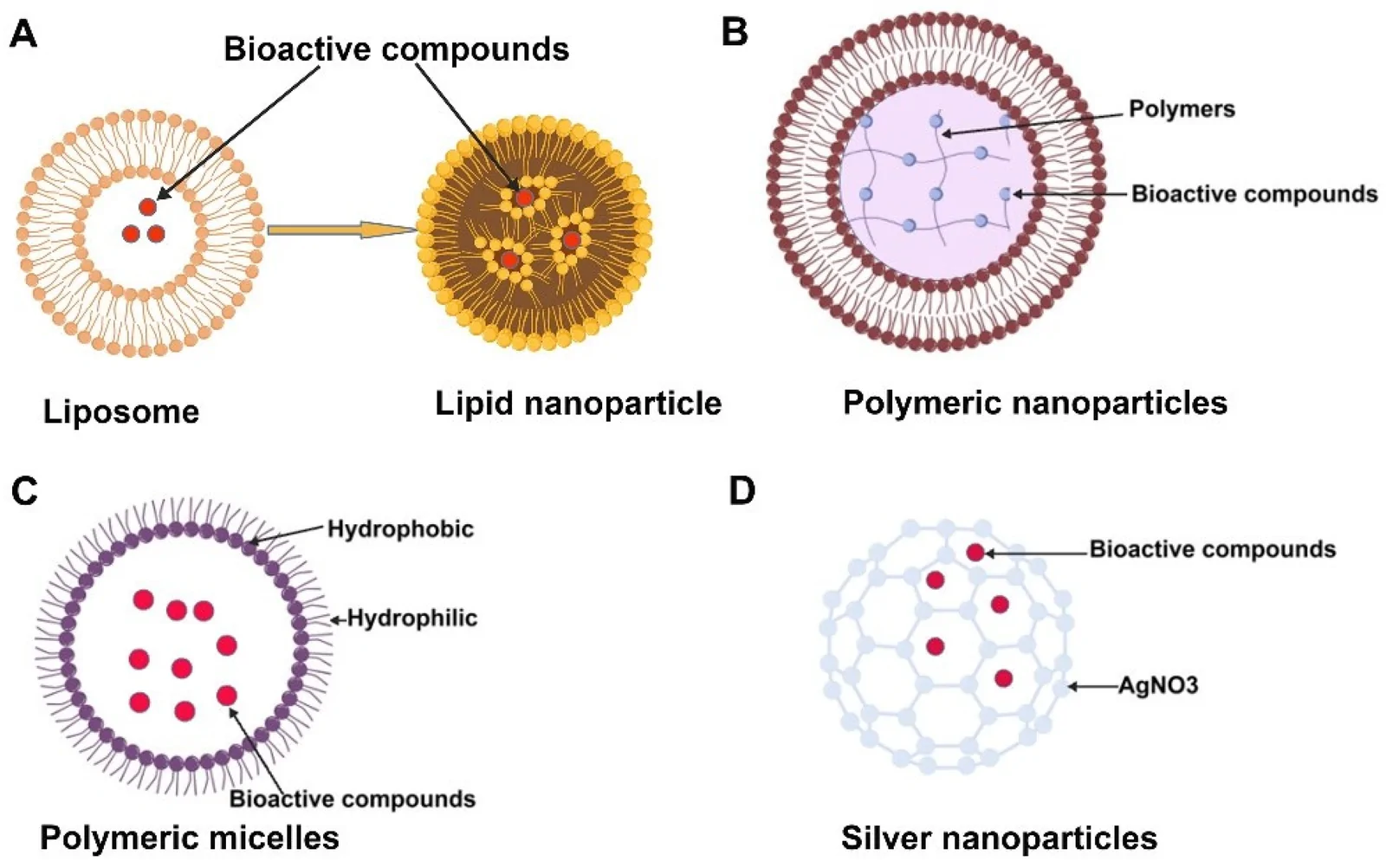
Schematic architectures of nanocarrier platforms for *G. lucidum* bioactive delivery. (**A**) Lipid-based carriers (liposomes, SLN, nanostructured lipid carriers) with bilayer (liposomes) or solid core (SLNs/NLCs) structures encapsulating lipophilic compounds. (**B**) Polymeric nanoparticles with bioactive compounds embedded in polymer matrix. (**C**) Polymeric micelles with hydrophobic core and hydrophilic shell for solubilizing poorly soluble compounds. (**D**) Silver nanoparticles (AgNPs) synthesized using *G. lucidum* extract as reducing and stabilizing agent. Blue and orange indicate hydrophilic and hydrophobic domains, respectively. All figures were created by the authors using Adobe Illustrator CC 2018 SP. None of the figures were taken from other articles.

For *G. lucidum* applications, the choice of platform is further complicated by the multiplicity of bioactive compounds. A formulation optimized for lipophilic triterpenoids may be suboptimal for hydrophilic GLPs. A formulation designed for intravenous administration may be unsuitable for oral delivery. The field has not yet grappled with whether a single “universal” nanocarrier can accommodate the full range of *G. lucidum* bioactives, or whether separate formulations will be required for different compound classes and indications. The emergence of multifunctional platforms such as GLP-LU-TeNRs and GLNs adds another dimension. Should the field pursue purified compound formulations with well-defined pharmacokinetics? Or complex assemblies that preserve the holistic character of the traditional preparation while offering improved delivery? Figure 2 provides a schematic comparison of the four principal nanocarrier platforms discussed in this section, highlighting their structural features and relative advantages for different *G. lucidum* cargo types. Table 3 compiles the nanoformulations surveyed in this section, listing cargo types, key findings, and limitations for each platform. The picture that emerges is one of isolated proof-of-concept studies—with little comparative data and no clinical translation.

## 5. Clinical Evidence: The Gap Between Preclinical Promise and Human Data

The preclinical literature on *G. lucidum* is vast, yet the clinical evidence base remains strikingly modest. Hundreds of in vitro and animal studies have reported bioactivity. The number of well-designed, adequately powered randomized controlled trials (RCTs), however, is small. The overall quality of existing data has been judged insufficient to inform clinical practice [16].

### 5.1. The Cochrane Review: A Critical Benchmark

The Cochrane systematic review constitutes the most comprehensive synthesis of clinical evidence for *G. lucidum* in oncology [16]. Its conclusion was unambiguous: the quality of primary studies was generally unsatisfactory, and no sufficient evidence supported *G. lucidum* as a first-line cancer therapy. The review identified recurrent methodological shortcomings—small sample sizes, lack of blinding, and inadequate allocation concealment. Pronounced heterogeneity in the tested preparations further complicated interpretation. Different trials used distinct extracts—polysaccharide-rich, triterpenoid-rich, or crude whole-powder formulations—at varying doses. This heterogeneity made it difficult to discern whether negative outcomes reflected an inherent lack of efficacy or simply an inadequate formulation of the specific product tested.

Nearly a decade later, this conclusion remains essentially unchanged. Not because the Cochrane reviewers failed to set a rigorous standard, but because the field has persistently declined to meet it. The same deficiencies—small samples, open-label designs, and disparate preparations—continue to pervade most post-2016 clinical studies of *G. lucidum*. The review did acknowledge potential immunomodulatory benefits when *G. lucidum* was used as an adjunct. These observations, however, derived from small, low-quality studies and did not achieve statistical significance in meta-analyses.

### 5.2. Clinical Studies Beyond Cancer

Outside oncology, clinical exploration has targeted type 2 diabetes, metabolic syndrome, and rheumatoid arthritis, among other conditions. Yet, here too, the pattern of equivocal outcomes recurs with striking consistency. Two recent meta-analyses have scrutinized the metabolic effects of G. lucidum supplementation in human populations. Their conclusions converge on a sobering assessment.

One meta-analysis—a systematic review of 13 studies covering metabolic syndrome, type 2 diabetes, fibromyalgia, and coronary artery disease—found no significant between-group differences in HDL, LDL, total cholesterol, or fasting plasma glucose among metabolic syndrome populations [13]. Within-group comparisons among healthy individuals revealed significant reductions only in serum glutamic-pyruvic transaminase and total cholesterol. These signals, though statistically detectable, remain clinically marginal and mechanistically unmoored [13].

The second meta-analysis—a GRADE-assessed systematic review of 17 RCTs (971 participants)—examined *G. lucidum* across doses ranging from 200 to 11,200 mg/day and treatment durations of 1 to 24 weeks [14]. The pooled analysis identified modest reductions in BMI, creatinine, and heart rate, alongside an increase in glutathione peroxidase. These effects reached statistical significance but were rated as very low certainty across all outcomes [14].

These meta-analyses paint a consistent picture. They detect statistically significant shifts in secondary endpoints. Yet, they find no reliable, clinically meaningful changes in primary metabolic targets that would justify therapeutic recommendation. The heterogeneity of preparations, doses, and populations across the included trials undercuts any robust synthesis. The absence of standardized *G. lucidum* extracts—a recurring theme in this review—only deepens the interpretive uncertainty. The collective message is not that *G. lucidum* is metabolically inert. Rather, any activity it may possess is likely diluted—by the very bioavailability constraints that define the central problem of this field.

A double-blind, randomized, placebo-controlled trial of *G. lucidum* (3 g/day) in individuals with type 2 diabetes and metabolic syndrome reported no significant adverse effects. Some favorable shifts in cardiovascular risk markers were observed, though the clinical magnitude was modest [81]. A randomized trial of *G. lucidum* extract in men with lower urinary tract symptoms noted significant improvement in the International Prostate Symptom Score [82]. By contrast, a 2025 systematic review of clinical trials examining *G. lucidum* supplementation and blood lipids found that only 2 of 7 trials demonstrated a moderate decrease in total cholesterol. The authors concluded that no evidence supports a lipid-modulating effect [83]. Separately, a randomized crossover study in healthy male volunteers evaluated whether oral coadministration of ascorbic acid with a Lingzhi preparation affected the pharmacokinetics of ganoderic acids. Ascorbic acid did not significantly alter their pharmacokinetic parameters [84].

This diversity of indications reflects both the breadth of *G. lucidum*’s reported bioactivities and the fragmentation of the research enterprise. The field has pursued numerous potential applications without achieving definitive results in any single one. This scattershot strategy has generated a voluminous literature. It has not, however, produced the focused, high-quality evidence necessary for regulatory endorsement or clinical uptake. No indication has accumulated sufficient evidence to meet the threshold for clinical recommendation. This shortfall stems directly from the field’s failure to concentrate resources on its most promising leads.

### 5.3. The Missing Link: Clinical Studies of Nanoformulations

The most conspicuous void in the clinical evidence base is the total absence of human trials for *G. lucidum* nanoformulations. Despite encouraging preclinical data, no such formulation has advanced to clinical testing [2]. This gap does not signify a scientific impasse. Rather, it reflects the nascency of the field. Nanocarrier formulations for natural products face substantial regulatory, manufacturing, and funding obstacles.

The regulatory pathway for *G. lucidum* nanoformulations remains undefined. Unlike single-agent synthetic drugs, *G. lucidum* extracts contain dozens of bioactive compounds. Each compound may partition differently within a nanoparticle matrix. This compositional heterogeneity complicates the establishment of meaningful quality-control standards. It also obscures which components are responsible for therapeutic activity [85]. Regulatory agencies, accustomed to evaluating well-characterized single entities, have limited experience with complex natural-product nanomedicines. The emergence of multifunctional platforms—such as GLP-LU-TeNRs and GLNs—further complicates the landscape. These materials do not fit neatly into established categories of “drug,” “device,” or “biologic.”

Manufacturing scalability presents an additional barrier. Most *G. lucidum* nanoformulations have been developed at the laboratory scale using batch processes that are not readily transferable to industrial production [64]. Batch-to-batch variability inherent in natural-product extraction compounds this challenge. Even if a nanocarrier formulation is optimized for one extract batch, the next batch may exhibit a different compositional profile, necessitating re-optimization. Without standardized starting materials and validated manufacturing protocols, regulatory approval remains out of reach.

**Figure 3:**
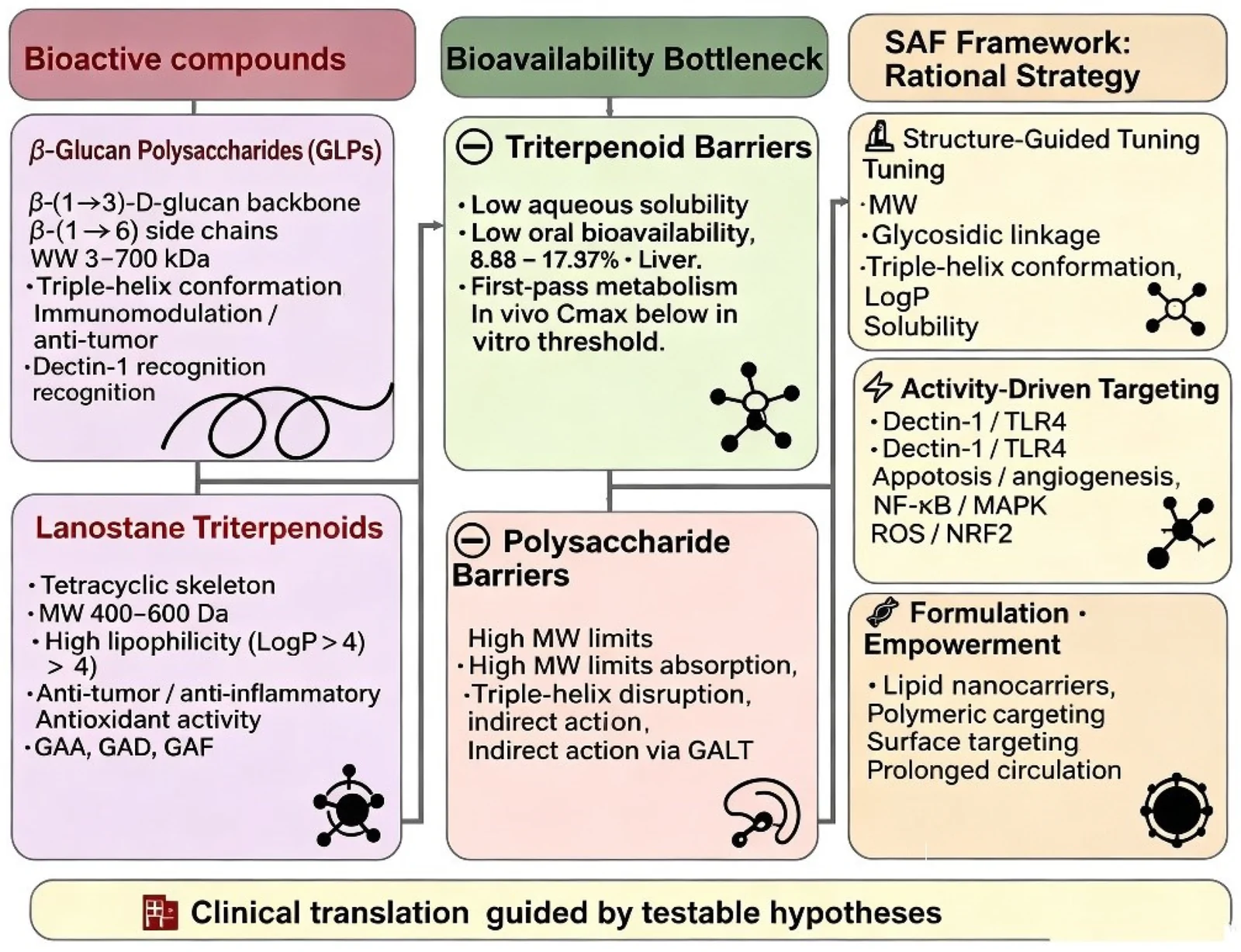
Structure–Activity–Formulation (SAF) framework for *G. lucidum* development. (**Left**): Bioactive compounds (GLPs and triterpenoids) with key structural features. (**Middle**): Bioavailability bottlenecks—poor solubility, rapid metabolism, and high molecular weight limit systemic exposure. (**Right**): The SAF framework integrates structural determinants, pharmacological activities, and formulation strategies to overcome delivery barriers. (**Bottom**): Clinical translation guided by testable hypotheses. BA, bioavailability; GAA, ganoderic acid A; GALT, gut-associated lymphoid tissue. All figures were created by the authors using Adobe Illustrator CC 2018 SP. None of the figures were taken from other articles.

The foregoing analysis across chemistry, extraction, formulation, and clinical domains exposes a unified failure pattern—not of pharmacological potency, but of delivery. To convert this recognition into a testable development roadmap, we consolidate these disconnected variables into a SAF framework (Figure 3). This framework anchors formulation design explicitly to the physicochemical determinants of bioactivity.

## 6. The SAF Framework: From Chemical Complexity to Rational Delivery

The preceding sections dissect the structural basis of *G. lucidum* bioactivity, the technical limitations of conventional extraction, and the translational bottlenecks confronting nanocarrier platforms. What emerges is a disconnected literature—one rich in isolated observations but devoid of an organizing principle. We propose the SAF framework as that unifying principle. It explicitly links chemical features, processing history, and delivery design to anchor a rational path toward clinical development.

### 6.1. Defining the SAF Framework

The SAF framework rests on three interdependent determinants of therapeutic success: chemical structure, which dictates intrinsic activity and physicochemical behavior; extraction and processing, which determine structural preservation and compositional consistency; and formulation strategy, which governs pharmacokinetic fate and, ultimately, in vivo efficacy.

These variables do not operate in isolation. Structure constrains the permissible extraction window—harsh conditions degrade labile conformations. Formulation must be tailored to the extracted material’s solubility, stability, and molecular size. Extraction choices shape which structural features remain available for formulation. Delivery systems can only partially compensate for deficiencies incurred upstream. Although recent reviews have systematically cataloged how extraction parameters affect polysaccharide integrity and bioactivity [12,86,87], the field has largely failed to integrate these discrete observations into a coherent, hypothesis-driven pipeline.

The SAF framework compels a strategic reordering of research priorities. Rather than pursuing all compounds, all extraction protocols, and all formulation vehicles in parallel, the field must concentrate on the intersecting subset most likely to yield therapeutic return. That subset comprises molecules with validated structureactivity relationships, processed under conditions that preserve their bioactive architecture, and encapsulated in carriers optimized for their specific physicochemical liabilities.

Consider a hypothetical scenario that is almost certainly playing out in the published literature without acknowledgment. A researcher isolates a potent ganoderic acid derivative. It is extracted under aggressive conditions that disrupt its active conformation. It is then encapsulated in a carrier unsuited to its lipophilicity. The result—negligible efficacy—is attributed to the compound itself. In fact, the failure lies entirely in the chain of handling and delivery. This misattribution is not an isolated risk. It is a systemic flaw that the SAF framework explicitly forces into view.

### 6.2. SAF Applied to Polysaccharides (GLPs)

Applying the SAF logic to GLPs dictates a clear imperative. Prioritize high-molecular-weight β-glucans with defined branching patterns. Extract them under mild conditions that preserve the triple-helix conformation. Formulate them in polymeric nanoparticles or liposomes that shield against degradation while facilitating uptake by immune cells. The structural determinants of Dectin-1 engagement—the β-(1→3) backbone, β-(1→6) side chains, and triple-helix architecture—must be tracked as critical quality attributes throughout extraction and formulation. GLP bioactivity is modulated by a constellation of interrelated parameters, including monosaccharide composition, glycosidic linkage profile, branching degree, and molecular weight distribution [86].

Formulation design should aim to deliver the polysaccharide to gut-associated lymphoid tissue (for oral routes) or to antigen-presenting cells in target tissues (for parenteral routes). Pursuing systemic circulation of the intact macromolecule is biophysically implausible for high-molecular-weight polymers. The extraction method itself—whether hot water, ultrasound, microwave, or enzyme-assisted—influences not merely yield but also the preservation of molecular weight distribution and conformational state [12,88]. Whether yield optimization inadvertently compromises bioactivity remains an open question. The SAF framework insists that this trade-off can no longer be ignored.

### 6.3. SAF Applied to Triterpenoids

For ganoderic acids and related triterpenoids, the SAF framework compels a shift from indiscriminate screening to targeted prioritization. Select compounds with the most favorable balance of potency and pharmacokinetic tractability. Extract them using methods that preserve structural integrity—supercritical CO_2_ or mild ethanol extraction. Formulate them in lipid nanoparticles or polymeric micelles to enhance solubility, attenuate first-pass metabolism, and promote tumor accumulation via the EPR effect. The high lipophilicity of triterpenoids makes them natural substrates for lipid-based carriers.

The sheer structural diversity of *Ganoderma* triterpenoids—nearly 500 compounds identified across 25 species—presents both an opportunity and a daunting prioritization challenge [30,33]. The pharmacokinetic profile of GAA provides clear target specifications for formulation. Its oral bioavailability ranges from 8.68% to 17.97%. It exhibits rapid absorption (Tmax < 0.611 h) and a short half-life (2.183–2.485 h). Any viable carrier must extend circulation, reduce hepatic clearance, and achieve sustained therapeutic concentrations [6,7]. A comprehensive review of GAA pharmacology confirmed its potent anti-inflammatory, antioxidant, and antitumor activities through NF-κB, JAK/STAT, TLR4, and MAPK pathways. Yet, clinical translation remains blocked by pharmacokinetic barriers rather than target engagement [3]. The improvement in GAD bioavailability from 22% to 70% upon SLN formulation offers quantitative proof of concept for what rational design can achieve [38]. Structure–activity relationships among ganoderic acids—wherein specific hydroxyl, carbonyl, and keto substitutions on the lanostane skeleton determine both potency and physicochemical properties—provide a rational basis for compound selection and prioritization [10,44].

### 6.4. Confronting Complexity: The Mixture Problem

Perhaps the most formidable challenge the SAF framework exposes is the inherent tension between natural-product complexity and formulation precision. Different constituents within a single extract may possess divergent structure–activity profiles and conflicting formulation requirements. If so, no single extraction or delivery strategy can optimally serve all. The field must therefore confront a strategic choice. One path leads toward purified, well-characterized single compounds or defined mixtures that offer reproducibility, mechanistic clarity, and regulatory feasibility. The other embraces the holistic complexity of the natural matrix while engineering delivery at the supramolecular level.

The emergence of multifunctional platforms such as GLP-LU-TeNRs and GLNs suggests a third path. This approach preserves compositional complexity while addressing bioavailability through nanoscale assembly. It retains the potential for synergistic interactions among multiple bioactive constituents. Yet, it does not imply a one-size-fits-all carrier. Rather, it demands rational co-encapsulation of specific triterpenoid–polysaccharide pairs with demonstrated synergy (see Hypothesis 3). It also concedes that different indications and compound classes will likely require tailored, indication-specific systems. Whether this complexity-oriented strategy can achieve regulatory acceptance remains an open question. Early engagement with agencies on quality-by-design principles may preempt some of the uncertainty.

What is no longer tenable is the field’s current default: randomized screening without structural oversight, extraction without integrity monitoring, and formulation without pharmacokinetic rationale. The SAF framework does not solve these problems. It makes them unavoidable—and that is its value. The SAF framework’s application to GLPs and triterpenoids is distilled in Table 4, which links structural features to activity and formulation strategy, with quantitative success criteria for each class.

## 7. Testable Hypotheses and Future Directions

The preceding analysis yields several testable hypotheses.

**Hypothesis** **1.** *Lipid nanoparticle encapsulation of GAA will increase its oral bioavailability by at least 5-fold relative to free-drug administration in a rat model. This will be measured by area under the concentration-time curve (AUC) and maximum concentration (Cmax). Pharmacokinetic methods for testing this prediction are well-established [6,7]. A quantitative precedent already exists. The absolute bioavailability of GAD increased from 22% to 70% upon SLN encapsulation [38]—a >3-fold enhancement. That enhancement was achieved without systematic optimization of lipid composition or surface modification. Given that GAA’s intrinsic bioavailability is lower (8.68–17.97%), a 5-fold improvement (targeting ~43–90%) represents a conservative and readily testable benchmark. Such an improvement would bring GAA exposure closer to therapeutic concentrations.*

**Hypothesis** **2.** *GLPs extracted under mild conditions that preserve the triple-helix conformation will exhibit greater immunomodulatory activity in vitro and in vivo than those extracted under harsh conditions that denature this structure. This difference should hold even at lower yields. This hypothesis directly addresses whether yield optimization or bioactivity preservation should guide extraction method selection. Testing it would require systematic comparison of extracts prepared by different methods. It would also require structural characterization—molecular weight, conformation, and branching pattern—and bioactivity assessment in standardized assays.*

**Hypothesis** **3.** *Co-delivery of ganoderic acids and GLPs within a single nanoparticle formulation will produce synergistic antitumor effects in a mouse xenograft model. These effects should exceed the sum of effects from separate formulations. This hypothesis asks whether the traditional use of whole extracts has a pharmacological basis that nanotechnology can preserve and enhance. Mechanistic support comes from reported synergistic anti-inflammatory effects of a specific G. lucidum polysaccharide fraction (designated GLP-1 in the cited study) and GAA through co-targeting of TLR4/NF-κB [89]. Testing this hypothesis would require development of a co-delivery platform and direct comparison with mono-delivery formulations.*

These hypotheses are mechanistically grounded and testable with currently available methods. Collectively, they address the central question animating this review: how can the field move from phenomenological description to hypothesis-driven investigation?

## 8. Conclusions

The SAF framework formalizes a reality the field has long acknowledged but rarely acted upon. Structural features that confer activity inherently impose pharmacokinetic penalties. The choice of extraction method determines not just yield but whether bioactive conformations survive for formulation. The extraordinary pharmacological breadth of *G. lucidum* stands in stark contrast to its limited clinical footprint. This disconnect reflects a failure of delivery, not a failure of chemistry.

Recent advances in understanding the pharmacokinetic diversity of individual ganoderic acids underscore both the severity of the intrinsic delivery challenge and the potential of rational formulation to overcome it. Free compounds typically exhibit oral bioavailabilities below 20%—8.68–17.97% for GAA [6,7]. Yet, encapsulation can dramatically elevate these values. GAD reached 70% upon SLN formulation [38]. That such dramatic enhancement depends entirely on sophisticated carrier systems reinforces our central thesis. Nanotechnology is not an optional add-on. It is a fundamental prerequisite for unlocking the therapeutic potential of these compounds.

The field must now do several things differently.

First, it can no longer afford the phenomenological enumeration paradigm that has dominated its literature. Exhaustive cataloging of compounds and activities has generated a broad foundation, but it has also obscured the need for mechanistic clarity and quantitative standards.

Second, bioavailability must be treated as a primary design parameter, not an optional enhancement. Extraction must be optimized for bioactivity retention, not merely for mass recovery. Nanocarriers must be benchmarked against pharmacokinetic endpoints rather than cytotoxicity assays alone.

Third, without purified, structurally validated reference materials and scalable, Good Manufacturing Practice (GMP)-compliant manufacturing, even the most elegant nanoformulations will never reach a patient.

Fourth, and perhaps most critically, the economic reality: without patent protection on the natural starting material, pharmaceutical companies have little commercial incentive to invest in costly clinical development of *G. lucidum* nanoformulations. This economic reality, not scientific uncertainty, may ultimately determine whether these promising formulations reach patients—a point the scientific literature has been notably reluctant to acknowledge.

Beyond the biological and regulatory hurdles stands a formidable economic bottleneck. *G. lucidum*’s fundamental composition—its triterpenoid skeleton and glucan backbone—is a naturally occurring, non-patentable entity. Without composition-of-matter patent protection, the multi-million-dollar investment required for Phase II/III trials presents pharmaceutical corporations with a starkly negative risk–reward calculus. That is why, despite decades of promising preclinical data, no major pharma company has advanced a *G. lucidum* formulation into late-stage development.

Patent landscape analyses confirm the pattern: the global technological evolution of *Ganoderma* mushrooms has focused predominantly on cultivation methods, extraction processes, and fermentation strains. Novel therapeutic formulations or delivery systems have not been the priority [90].

The path forward cannot lie in futile attempts to patent the natural molecule. Strategic intellectual property must instead surround novel formulation architectures, specific carrier-to-cargo ratios, and indication-specific co-delivery synergies (e.g., Hypothesis 3). Without a deliberate IP strategy, even the most elegant SAF-driven design will perish in the gap that separates academic demonstration from commercial reality.

The challenges are significant but not unprecedented. The nanocarrier platforms that have succeeded for other poorly soluble drugs—liposomes, polymeric nanoparticles, and lipid nanoparticles—offer proven engineering templates. Regulatory pathways for complex natural-product nanomedicines remain uncharted. Early engagement with agencies on quality control and manufacturing standards can reduce uncertainty. The path forward demands a deliberate shift from description to mechanism, from cataloging to solving. The ancient remedy has been revered for its potential. Modern therapeutics demand more than reverence. They demand a rigorous, hypothesis-driven delivery strategy—one that is now within reach, provided the field pursues it with the rigor it deserves.

## Figures and Tables

**Table 1:** Pharmacokinetic parameters of representative *G. lucidum* triterpenoids after oral administration.

Compound	Dose/Formulation	Species	BA (%)	Cmax (ng/mL)	Tmax (h)	t1/2 (h)	Key Findings	Ref.
GAA	100 mg/kg, p.o.	Rat	8.68	358.7	0.611	2.48	Brain-to-plasma ratio 0.05–0.18	[6]
GAA	400 mg/kg, p.o.	Rat	17.97	3010.4	0.24	2.18	Dose-dependent increase in exposure	[7]
GAD	Free drug, p.o.	Rat	22	107.2	2.0	NR	Baseline oral PK	[38]
GAD	GAD-loaded SLN, p.o.	Rat	70	1555.6	0.3	NR	SLN formulation enhanced BA and Cmax	[38]
GAF	Lingzhi preparation, p.o	Human	NR	NR (decreased with food)	~0.5 (fasted)	<0.7	Food significantly decreased Cmax and delayed Tmax	[37]

Abbreviations: BA, bioavailability; SLN, solid lipid nanoparticle; NR, not reported.

**Table 2:** Comparison of extraction methods for G. lucidum bioactives: yield vs. structural integrity and bioactivity.

Method	Target	Reported Yield	Effect on Structural Integrity	Key Limitation
Hot water extraction (HWE)	GLPs	~1.5%	May denature triple-helix [29]; alkaline conditions may hydrolyze glycosidic bonds [47]	Low yield; potential activity loss [29,46,47].
Ethanol maceration	Triterpenoids	~0.6%	Preserves intact skeleton; co-extracts lipids [46]	Low yield; slow process [46]
Ultrasonic-assisted (UAE)	Triterpenoids + phenolics	4.9% recovery; 435.6 mg/g triterpenoids [48]	Prolonged sonication degrades GLPs (chain scission) [20]	No immunomodulatory/antitumor bioactivity data; only antioxidant activity reported [20,48]
Microwave-assisted (MAE)	GLPs + triterpenoids	13.08 mg glucose/g GLPs; 9.15 mg ursolic acid/g triterpenoids [50]	Thermal degradation risk; effect on triple-helix unknown [50]	No bioactivity data [50]
Ultrasonic-microwave (UMAE)	GLPs	115.6% over HWE; 27.7% over UAE [51]	May reduce Mw and alter conformation [51]	No bioactivity data [51]
Supercritical CO_2_ (SCE)	Triterpenoids	1.56 mg/100 g [52]	Preserves labile compounds (low temperature) [52]	Very low yield; high cost [52]

**Table 3:** Summary of nanoformulations for *G. lucidum* bioactive delivery.

Platform	Cargo	Key Findings	Key Limitations	Ref.
Lipid-based				
Zein-chitosan NPs (GA-NPs)	Ganoderic acids (crude)	Alcohol-induced liver injury mouse: improved liver function, reduced oxidative stress, ameliorated gut dysbiosis (superior to free drug)	Single disease model; no PK data; crude cargo	[43]
Solid lipid NPs (GAD-SLNs)	Ganoderic acid D (GAD)	Rat PK: BA ↑ 22%→70%; Cmax ↑ 107 → 1555.6 ng/mL; Tmax ↓ 2.0→0.3 h	No efficacy data; scalability not addressed	[38]
Solid lipid NPs (GA-SLNs)	Ganoderic acid (crude)	D-galactosamine hepatotoxicity rat: restored serum hepatic markers and antioxidant enzymes vs. free drug	Single disease model; no PK optimization	[58]
GLP liposomes	GLPs	Porcine circovirus mouse: enhanced antibody titers and cytokine production	Immunogenicity study only; no PK data	[60]
GLP-LNPs	GLPs + mRNA-LNP	Alleviated oxidative stress (GSH ↑, SOD ↑, MDA ↓); innate immune modulation	In vitro only; mechanism incompletely defined	[61]
Polymeric				
Self-assembled GLP NPs	GLPs	Cytotoxic to tumor cells; promoted spleen cell growth	In vitro only; no in vivo data	[65]
pH-sensitive GLP-MTX NPs	GLP + methotrexate	Programmable drug release; enhanced antitumor activity in xenografts	Complex synthesis; limited in vivo characterization	[66,67]
GLP-stabilized SeNPs (GLP-SeNPs)	GLPs + selenium	MAFLD mouse: prevented ferroptosis via NRF2/GSH-GPX4 and ACSL4 pathways	Selenium safety unknown; no PK data	[68]
Chitosan-polysaccharide NPs	GLPs	PC3 cells: *BAX* ↑ 4.6-fold, *BCL2* ↓ to 0.64-fold → apoptosis	In vitro only; no in vivo efficacy	[69]
Polymeric micelles	Ganoderic acids	Enhanced solubility and bioavailability of ganoderic acids (conceptual, based on GAD-SLN data)	Instability upon dilution; GI degradation; limited *G. lucidum*-specific data	[70,71]
Metallic				
GL-AgNPs	AgNO_3_+ extract	Antimicrobial activity against drug-resistant *E. coli*	Metal accumulation; off-target toxicity; low biodegradability	[72,73,74]
Emerging				
GLP-LU-TeNRs	GLPs + luteolin + Te	Photothermal (30.9%); in vitro inhibition 22.8%; in vivo tumor ↓ 36.1%, metastasis ↓ 66.7%	Laser conditions not clinically feasible; Te safety unknown	[76]
*G. lucidum* nanovesicles (GLNs)	Endogenous lipids/proteins/RNAs	Oral: reduced ALT/AST, MDA ↓, Nrf2/HO-1 ↑, NF-κB ↓, IL-6/TNF-α ↓, IL-10 ↑	Human BA speculative; mechanism incompletely defined	[77]
Topical				
Dissolvable microneedle (MN-WSG)	Water-soluble glucan (WSG)	B16F10 melanoma mouse: suppressed tumor growth via TGFβ/Snail/Twist	Transdermal route; not oral; patch scalability	[78]

Abbreviations: BA, bioavailability; NP, nanoparticle; PK, pharmacokinetic; GLP, *G. lucidum* polysaccharide; SLN, solid lipid nanoparticle; LNP, lipid nanoparticle; SeNP, selenium nanoparticle; TeNR, tellurium nanorod; GLN, *G. lucidum*-derived nanovesicle; WSG, water-soluble glucan; MAFLD, metabolic-associated fatty liver disease; MTX, methotrexate. ↑, indicates an increase; ↓, indicates a decrease.

**Table 4:** Structure–Activity–Formulation (SAF) framework for *G. lucidum* bioactives.

Compound Class	Key Structural Features	Key Activity	Formulation Strategy	Critical Quality Attribute
GLPs	β-(1→3)-D-glucan backbone with β-(1→6)-branches; triple-helix conformation; Mw 3–700 kDa	Immunomodulation (Dectin-1/TLR4); antitumor (↑ CD4^+^/CD8^+^); anti-inflammatory; antioxidant	Mild extraction (preserve triple-helix); polymeric NPs or liposomes for GALT/APC targeting	Triple-helix retention > 80%; consistent Mw and composition; Dectin-1 binding (EC_50_)
Triterpenoids	Lanostane skeleton; –OH/–COOH/C=O substituents; Mw 400–600 Da; LogP > 4; BCS II/IV	Antitumor (apoptosis, anti-angiogenesis); anti-inflammatory (NF-κB, JAK/STAT, TLR4); antioxidant; neuroprotective	Lipid carriers (LNP/SLN/NLC) for BA enhancement; polymeric micelles; co-delivery with GLPs for synergy	Oral BA ≥ 5× over free drug; Cmax in micromolar range; t_1_/_2_ > 4 h; EPR-mediated tumor accumulation

Abbreviations: ↑, indicates an increase.

## Data Availability

No new data were created or analyzed in this study. Data sharing is not applicable to this article.

## Abbreviations

The following abbreviations are used in this manuscript:

ACSL4	acyl-CoA synthetase long-chain family member 4
ALT	alanine aminotransferase
AMPK	AMP-activated protein kinase
APC	antigen-presenting cell
AST	aspartate aminotransferase
AUC	area under the concentration-time curve
BA	bioavailability
*BAX*	*BCL2*-associated X protein
*BCL2*	B-cell lymphoma 2
BCS	Biopharmaceutics Classification System
CD206	mannose receptor
CNS	central nervous system
Cmax	maximum plasma concentration
EC50	half-maximal effective concentration
EPGLa	*G. lucidum* polysaccharide a
EPR	enhanced permeability and retention
GAA	ganoderic acid A
GAB	ganoderic acid B
GAC_1_	ganoderic acid C1
GAC_2_	ganoderic acid C2
GAD	ganoderic acid D
GAF	ganoderic acid F
GAG	ganoderic acid G
GAH	ganoderic acid H
GAK	ganoderic acid K
GALT	gut-associated lymphoid tissue
GA-NPs	ganoderic acid-loaded nanoparticles
GA-SLNs	ganoderic acid-loaded solid lipid nanoparticles
GL-AgNPs	*G. lucidum*-mediated silver nanoparticles
GLNs	*G. lucidum*-derived nanovesicles
GLP	*G. lucidum* polysaccharide
GLP-LNPs	GLP-loaded lipid nanoparticles
GLP-LU-TeNRs	GLP-based multifunctional tellurium nanorods
GLP-MTX	GLP-methotrexate conjugate
GLP-SeNPs	GLP-stabilized selenium nanoparticles
GMP	Good Manufacturing Practice
GSH-GPX4	glutathione-glutathione peroxidase 4
HO-1	heme oxygenase-1
HPLC	high-performance liquid chromatography
HWE	hot water extraction
IL-6	interleukin-6
JAK/STAT	Janus kinase/signal transducer and activator of transcription
LC-MS/MS	liquid chromatography-tandem mass spectrometry
LNP	lipid nanoparticle
MAE	microwave-assisted extraction
MAFLD	metabolic-associated fatty liver disease
MAPK	mitogen-activated protein kinase
MN-WSG	dissolvable microneedle patch loaded with water-soluble glucan
mRNA	messenger RNA
Mw	molecular weight
NF-κB	nuclear factor kappa-B
NLC	nanostructured lipid carrier
NO	nitric oxide
NP	nanoparticle
NRF2	nuclear factor erythroid 2-related factor 2
PK	pharmacokinetic
PLGA	poly(lactic-co-glycolic acid)
PNP	polymeric nanoparticle
RCT	randomized controlled trial
ROS	reactive oxygen species
SAF	structure–activity–formulation
SCE	supercritical CO_2_ extraction
SeNP	selenium nanoparticle
SLN	solid lipid nanoparticle
SOD	superoxide dismutase
t_1_/_2_	elimination half-life
TeNR	tellurium nanorod
TLR4	Toll-like receptor 4
Tmax	time to maximum plasma concentration
TNF-α	tumor necrosis factor-alpha
UAE	ultrasonic-assisted extraction
UMAE	ultrasonic-microwave-assisted extraction
UPLC-Q-TOF/MS	ultra-performance liquid chromatography quadrupole time-of-flight mass spectrometry
WSG	water-soluble glucan
XRCC1	X-ray repair cross-complementing protein 1
